# Editorial: New insights and advances in body recomposition

**DOI:** 10.3389/fnut.2024.1467406

**Published:** 2024-09-03

**Authors:** Diego A. Bonilla, Jorge L. Petro, Roberto Cannataro, Richard B. Kreider, Jeffrey R. Stout

**Affiliations:** ^1^Research Division, Dynamical Business and Science Society–DBSS International SAS, Bogotá, Colombia; ^2^Research Group in Physical Activity, Sports and Health Sciences (GICAFS), Universidad de Córdoba, Montería, Colombia; ^3^Hologenomiks Research Group, Department of Genetics, Physical Anthropology and Animal Physiology, University of the Basque Country (UPV/EHU), Leioa, Spain; ^4^Department of Pharmacy, Health and Nutritional Sciences, University of Calabria, Rende, Italy; ^5^Galascreen Laboratories, University of Calabria, Rende, Italy; ^6^Exercise and Sport Nutrition Laboratory, Human Clinical Research Facility, Texas A&M University, College Station, TX, United States; ^7^Physiology of Work and Exercise Response (POWER) Laboratory, Institute of Exercise Physiology and Rehabilitation Science, University of Central Florida, Orlando, FL, United States

**Keywords:** body composition, physiological stress responses, skeletal muscle, nutritional physiological phenomena, sports nutrition, dietary supplements

Body recomposition is commonly defined as the simultaneous process of reducing body fat while maintaining or increasing lean mass, frequently with no changes in total body mass. It has gained popularity in the exercise and sports nutrition field, particularly in the fitness and bodybuilding sector. Although body recomposition is a relatively new term, the scientific world has examined this phenomenon for decades. Researchers have studied and developed strategies to reduce fat mass (FM) while preserving skeletal muscle mass (SMM) and resting energy expenditure (REE) through the implementation of different types of exercise and diet intervention programs—for example, for the prevention and management of obesity (1).

In particular, the Research Topic “*New Insights and Advances in Body Recomposition*” offers new contributions in the areas of body composition assessment, dietary interventions, and training recommendations for diverse populations. In the article “*Deuterium oxide validation of bioimpedance total body water estimates in Hispanic adults*,” Tinsley et al. validated the use of single-frequency BIA (Quantum V, RJL Systems, USA) and bioimpedance spectroscopy (SFB7 Impedimed, Australia) devices for the estimation of total body water using the Deuterium oxide dilution technique as reference in Hispanic adults. Importantly, the standard error of estimate and total error values were ≤ 2.3 L and Lin's concordance correlation coefficient were ≥0.96 for all comparisons.

From a nutritional perspective, rather than using a continuous and aggressive energy deficit, the application of a high-protein diet with intermittent and progressive energy restrictions plus resistance training (RT) might preserve fat-free mass (FFM) (2) and enhance dietary adherence (3) during body recomposition. The characteristics and effects of diet refeeds and diet breaks during a physique contest preparation are discussed elsewhere (4) albeit short-term benefits of the latter seems debatable in resistance-trained females (5). Creatine monohydrate is another valuable nutritional strategy that should be considered (6). What seems clear is the positive and protective effects of the high-protein diets on lean mass. In the article “*Effects of 8 weeks of resistance training in combination with a high protein diet on body composition, muscular performance, and markers of liver and kidney function in untrained older ex-military men*,” Bagheri et al. demonstrated that a daily protein intake of 1.6 g/kg/day is superior to 0.8 g/kg/day for promoting greater improvements in BIA-estimated SMM and 1-RM during a 8-week RT program in untrained older ex-military men (>60 years).

Interestingly, the findings reported in the article “*Effects of 8-week alkaline diet and aerobic exercise on body composition, aerobic performance, and lipid profiles in sedentary women*” showed the potential of alkaline foods to reduce markers associated with cardiometabolic risk (Yalcinkaya et al.). This dietary regimen is also called negative potential renal acid load (PRAL) and generally consist of diets rich in vegetables and fruits, alkali-rich, and low-phosphorus beverages. Although significant improvements were found in BMI, cardiovascular performance, and TG and c-LDL concentrations, the groups with the alkaline diet intervention failed to show improvements in body recomposition, possibly due to limited protein intake. Further clinical trials are needed in sedentary and other populations before drawing definitive conclusions on health metabolic protection. This is particularly important if we consider that available meta-analytic evidence does not support the potential detrimental effects of high-phosphate intake on bone health (7) or the use of alkaline diets to prevent calcium loss (8) or to protect bone health (9).

There is collective awareness among scientists and practitioners on the importance of optimizing fat loss through interventions that avoid generalized loss of muscle mass or physical function such as RT or high-protein diets. In fact, maintaining adequate skeletal muscle status (quantity, structure, and metabolic-endocrine function) is crucial for health and disease. Since body recomposition has been demonstrated to occur in untrained, trained and highly trained populations of different ages, exercise training strategies represent an active line of study to enhance the efficiency of individual/population adaptive processes. In this context, strength training, whether combined with other anti-sarcopenia strategies (such as a high-protein diet or creatine supplementation) or not, serves as a safe and effective approach to counteract the progression of sarcopenia. It does so by enhancing body composition (increasing muscle mass) and functionality, both of which are negatively impacted by aging (10). In the article “*Characteristics of resistance training-based protocols in older adults with sarcopenic obesity: a scoping review of training procedure recommendations*,” Silva et al. summarized easy-to-understand recommendations for detailing the characteristics of RT protocols prescribed for older adults with sarcopenic obesity. This not only may contribute to clinical practice but also provides insights and highlights gaps in literature to commission future research.
